# Food support for tuberculosis care: coverage, outcomes, and implementation lessons from the *Ni-kshay Mitra* scheme in India

**DOI:** 10.5588/pha.26.0031

**Published:** 2026-08-24

**Authors:** P. Chavda, R. Patel, K. Mehta, K.C. Prajitha, K. Davara, K. Shah, J. Tankariya, H. Nakshiwala, N. Panchal, S. Nayak, A.M.V. Kumar

**Affiliations:** 1 Department of Community Medicine, GMERS Medical College, Gotri, Vadodara, India;; 2 City Tuberculosis Office, Vadodara Municipal Corporation, Vadodara, India;; 3 International Union against Tuberculosis and Lung Disease (The Union), Paris, France;; 4 WHO Tuberculosis Technical Support Network, Vadodara, India;; 5 The Union South-East Asia Office, New Delhi, India;; 6 Yenepoya Medical College, Yenepoya (Deemed to be University), Mangaluru, India.

**Keywords:** urban health, vulnerable populations, programme evaluation, operational research, SORT IT

## Abstract

**SETTING:**

Vadodara City (Gujarat, India) has a mixed public–private tuberculosis (TB) care landscape. Since 2022, the city has implemented ‘*Ni-kshay Mitra*’ scheme, where individuals and organisations voluntarily ‘adopt’ persons with TB (PwTB) and provide food kits (distributed at monthly camps) during treatment.

**OBJECTIVE:**

To assess coverage (received at least one kit) and effectiveness of Ni-kshay Mitra on treatment outcomes, and explore implementation facilitators, barriers, and solutions from the stakeholders’ perspective.

**DESIGN:**

Explanatory mixed-methods study: quantitative cohort analysis of secondary programme data followed by qualitative interviews and focus group discussions with donors (n = 3), programme staff (n = 18), and PwTB (n = 35).

**RESULTS:**

Among 2,068 PwTB notified between January and June 2025, 1,238 (60%) consented to the scheme, 666 (32%) received ≥1 food kit, and 68 (3%) received complete package (≥6 kits). Median kits received were 2 (interquartile range: 2–4). Unsuccessful treatment outcomes were 7% (2022, pre-scheme) and 6% (2023, post-scheme), not statistically significant. Donor non-availability and opportunity costs of attending camps were the major barriers; presence of dedicated non-profit organisations was a key facilitator.

**CONCLUSION:**

Coverage of *Ni-kshay Mitra* remains suboptimal, largely due to donor participation gaps, possibly explaining limited effectiveness on outcomes. Strengthening donor engagement and alternate delivery models are essential to enhance reach and effectiveness.

## Graphical abstract

**Figure:**
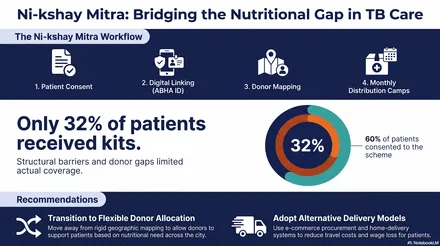

Tuberculosis (TB) remains one of the world’s most significant public health challenges. In 2024, an estimated 10.7 million people developed TB and 1.23 million died due to the disease, reaffirming its position as the leading infectious cause of death globally.^1^ India continues to bear the highest burden, contributing approximately one quarter of all TB cases worldwide.^1^ To accelerate progress towards ending TB, several strategic initiatives have been deployed by India, particularly those addressing undernutrition, a critical yet modifiable determinant of TB outcomes.

Undernutrition and TB reinforce each other in a cyclical manner. Undernutrition increases susceptibility to infection, risk of progression to active TB, delayed recovery, poor adherence to therapy, and unfavourable outcomes. Conversely, TB exacerbates undernutrition by increasing metabolic demands and reducing appetite. Evidence from programmatic settings in India consistently shows that food supplementation can support nutritional recovery and improve treatment outcomes among persons with TB (PwTB).^2,3^ Recognising this, the Government of India launched a series of nutritional support schemes, beginning with the Ni-kshay Poshan Yojana in 2018, which provides monthly financial support through bank transfer to all notified PwTB.^4^ In September 2022, the Government of India expanded its efforts and introduced ‘Ni-kshay Mitra’ (meaning ‘Friend of TB patient’) scheme. This initiative adopts a community-engagement model, inviting individuals and organisations to voluntarily ‘adopt’ PwTB and provide in-kind nutritional support.^5^ Registered *Ni-kshay Mitras* can include citizens, non-governmental organisations, community-based or faith-based groups, corporate entities, and political or social bodies.^6^ Each Mitra donates monthly food kits valued at a minimum of INR 800 (∼8 USD), consisting of nutrient-dense, locally acceptable items such as pulses, cereals, nuts, and oil. Donors plan the kits contents within the broad guidelines in consultation with the local TB programme officer. These kits aim to complement medical treatment and alleviate nutritional vulnerability during TB treatment.

Early analyses from the national Ni-kshay portal indicate variable uptake across states (ranging from 13% to 22%), with limited evidence on implementation processes, programme acceptability, and the perspectives of key stakeholders.^7^ Initial studies from selected districts in India report encouraging trends, including improved treatment outcomes among kit recipients and notable gains in community participation.^8–10^ However, concerns remain regarding confidentiality, donor availability, supply interruptions, and sustainability of support.^10^ Furthermore, information on the operational realities experienced by programme staff and donors is scarce, particularly in urban settings with large and diverse TB populations. Gujarat has been among the better-performing states in terms of *Ni-kshay Mitra* enrolment,^7^ yet no systematic evaluation has been conducted to understand coverage, treatment outcomes, and implementation challenges within the state. Vadodara City, with its mixed public–private health system and substantial TB caseload (∼4,000 PwTB notified annually), presents an important setting for such an assessment.

Given these gaps, we undertook a mixed-methods operational research study to: 1) assess coverage of *Ni-kshay Mitra* scheme and examine its relationship with TB treatment outcomes in Vadodara City and 2) understand the facilitators, barriers, and potential solutions for effective implementation from the perspectives of patients, programme staff, and donors. This evidence aims to inform improvements to the scheme and bolster India’s broader TB elimination efforts.

## METHODS

We conducted an explanatory, mixed-methods study: quantitative phase (cohort study involving analysis of secondary data) followed by a qualitative phase (descriptive study).

### Study setting

Vadodara is the third-largest city in Gujarat, with a population of approximately 2.2 million.^11^ Nearly one fifth of residents live in slum or slum-like settlements, and health care services are delivered through a mix of public and private facilities.^11^ The Vadodara Municipal Corporation manages local public health services, including 40 Urban Primary Health Centres (UPHCs), 4 zonal hospitals providing secondary care, and 2 medical colleges with associated public hospitals. A robust private health care sector provides additional outpatient, inpatient, and specialty services.

### TB programme infrastructure

TB services in Vadodara are coordinated by the City TB Office under the National Tuberculosis Elimination Programme. The city has eight TB units, supported by senior treatment supervisors (STSs) and TB health visitors (TB-HVs). There are 21 Designated Microscopy Centres and multiple rapid molecular testing laboratories which provide diagnostic coverage. Standard treatment for drug-sensitive TB consists of a 6-month regimen (2 months of HRZE [isoniazid, rifampicin, pyrazinamide, and ethambutol] followed by 4 months of HR), provided free of cost. The programme undertakes a range of public health actions, including management of co-morbidities, social support, and follow-up of patients receiving treatment in both public and private sectors.

### Implementation of *Ni-kshay Mitra* in Vadodara municipal corporation

The *Ni-kshay Mitra* scheme was launched to mobilise community-based donors to provide in-kind nutritional support to PwTB (Figure 1). Donors include non-government organisations (NGOs), civil society groups (such as Lions Club and Rotary Club), corporate social responsibility (CSR) units, elected representatives, and individual citizens. Donors are registered on the Ni-kshay portal (an online TB programme management information system) and typically adopt PwTB linked to specific UPHCs.

**FIGURE 1.**
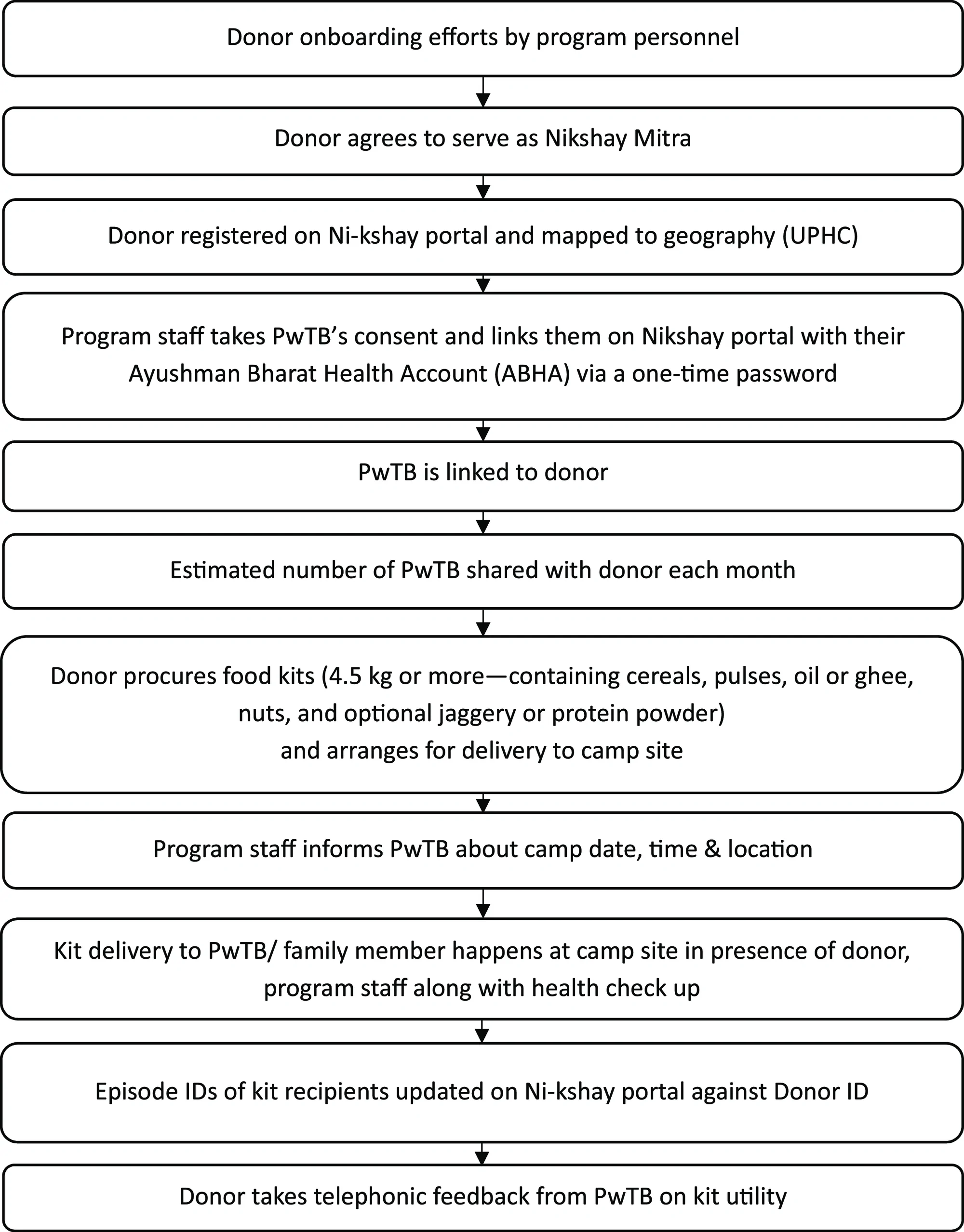
Flow chart depicting the *Ni-kshay Mitra* Programme Workflow in Vadodara City, 2025. UPHC = Urban Primary Health Centre; PwTB = persons with TB.

TB-HVs obtain consent from eligible PwTB after notification. The portal links the PwTB with Ayushman Bharat Health Account (ABHA) – a digital health record system for all citizens of India. Once a one-time password–based digital confirmation is provided, the patient is formally linked to a donor on the Ni-kshay portal. Donors procure and distribute monthly food kits – usually 4.5 kg or more – containing cereals, pulses, oil or ghee, nuts, and optional supplementary items such as jaggery or protein powder.

UPHCs organise monthly camps where kits are distributed to PwTB or a family member in the presence of donors and health care staff, and patients are also offered routine check-ups. Delivery details are first recorded on paper and later updated digitally on the Ni-kshay portal.

### Quantitative study population

This analysis included two sets of cohorts: coverage cohort (2025) and treatment outcomes cohort (2022 vs. 2023). All drug-sensitive PwTB notified between January and June 2025 in Vadodara City and initiated on first-line treatment were included in coverage cohort. Patients not on initiated treatment were excluded, as they would not be eligible for linkage to the scheme. Two additional cohorts of drug-sensitive PwTB started on first-line treatment were analysed to assess changes in treatment outcomes before (January–June 2022) and after (January–June 2023) the implementation of *Ni-kshay Mitra*.

### Qualitative study population

Three groups were purposively sampled: PwTB, programme staff, and donors. In-depth interviews (IDIs) were conducted with PwTB consented (n = 22) and non-consented (n = 13) to receive food kits, using maximum-variation sampling across age, gender, and public or private treatment sector (18 men, 17 women, age range 13–75 years). There were three and four non-responses among consenting and non-consenting PwTB, respectively. Health care providers who were knowledgeable, vocal, and actively involved in implementation were purposively selected (n = 18, 17 men, 1 woman, age range 30–56 years). One focus group discussion (FGD) each was conducted with STSs (n = 8) and TB-HVs (n = 6) cadres. IDIs were conducted with a City TB Officer and three medical officers of UPHCs. Three IDIs were conducted with donors active during the study period: two with NGO representatives and one with CSR manager.

### Data collection, study variables, and data sources

Quantitative: The data needed were extracted from three registers on the Ni-kshay portal: the notification register, the co-morbidity register, and the *Ni-kshay Mitra* register. For the 2025 cohort, variables included demographic and clinical characteristics, consent status for *Ni-kshay Mitra*, and number of kits received. For the 2022 and 2023 cohorts, treatment outcome data were additionally extracted. Qualitative: IDIs and FGDs were conducted by PC (Male, MD) and KD (Female, MD) who are trained medical faculty fluent in Gujarati and experienced in qualitative research. Participants were approached either in person or by telephone, and interviews were arranged at locations convenient to them ensuring sufficient privacy. Interviews lasted 25–30 min and were audio-recorded with consent. The interviews were conducted after quantitative data analysis, and findings of quantitative phase guided the qualitative exploration. Interview and FGD guides were adapted iteratively based on emerging qualitative findings. Recordings were transcribed verbatim in Gujarati and translated into English. Interviewers summarised key points at the end of each IDI/FGD to the participant/s to validate the information. Availability of participants and saturation of information guided the endpoint of data collection.

### Data analysis

Quantitative data cleaning and statistical analysis were conducted using STATA version 16. Coverage was operationally defined as ‘proportion receiving at least one food kit’. Modified Poisson regression with robust variance was used to estimate unadjusted relative risk (RR) and adjusted RR with 95% confidence intervals, adjusting for demographic, clinical, and behavioural variables to identify the factors associated with coverage. To compare treatment outcomes between 2022 and 2023 cohorts, treatment success was defined as cured or treatment completed. Multivariable regression (modified Poisson with robust variance) was used to assess if *Ni-kshay Mitra* was associated with unsuccessful treatment outcomes (treatment failure, loss to follow-up, died, not evaluated) after controlling for demographic, clinical, and behavioural variables. Qualitative: Manual content analysis was performed using a hybrid inductive–deductive approach. Codes related to facilitators, barriers, and implementation processes were generated independently by two investigators (PC and KD) and harmonised through discussion (KM, AKMV, KCP). Codes were grouped into themes, and findings were interpreted in relation to quantitative results. Reporting followed the Consolidated Criteria for Reporting Qualitative Research (COREQ) guidelines.^12^

### Ethical statement

The study received approval by the Institutional Ethics Committee (IHEC/25/0UT/FRO028, dated 5 August 2025). Written informed consent was obtained from all participants involved in the qualitative component. Permissions to access programme data were granted by the City TB Officer, the Chief Health Officer at Vadodara Municipal Corporation, and the State TB Officer.

## RESULTS

There were 2,068 PwTB notified between January and June 2025 and their demographic and clinical characteristics are summarised in Table 1.

**TABLE 1.** Demographic and clinical characteristics of persons with drug-sensitive tuberculosis (TB) notified and started on first-line treatment between January and June 2025 in Vadodara City, Gujarat, India.

Characteristics	n (%)
Total	2,068 (100)
Age (in years)	
0–14	70 (3)
15–29	660 (32)
30–44	540 (26)
45–59	434 (21)
60 and above	364 (18)
Gender	
Men	1,136 (55)
Women	930 (45)
Transgender	2 (<1)
BMI^A^	
Underweight (<18.5 kg/m^2^)	936 (45)
Normal (18.5–22.9 kg/m^2^)	903 (44)
Overweight/obesity (>23 kg/m^2^)	229 (11)
Notification	
Public sector	1,190 (58)
Private sector	878 (42)
Site of TB disease	
Pulmonary	1,309 (63)
Extra-pulmonary	745 (36)
Not recorded	14 (1)
Diagnosis of TB	
Bacteriologically confirmed	1,203 (58)
Clinically diagnosed	865 (42)
HIV	
Reactive	22 (1)
Non-reactive	1,989 (96)
Not recorded	57 (3)
Diabetes	
Yes	160 (8)
No	1,834 (89)
Not recorded	74 (3)
Current alcohol use	
Yes	231 (11)
No	1,730 (84)
Not recorded	107 (5)
Current tobacco use	
Yes	387 (19)
No	1,592 (77)
Not recorded	89 (4)

A BMI = body mass index (kg/m^2^), calculated as weight (kg) divided by height (m^2^) for participants aged ≥20 years. For participants aged <20 years, nutritional status was assessed using BMI-for-age Z-scores calculated using WHO age- and sex-specific growth standards and clubbed as follows: BMI < −2 SD as underweight, –2 SD to +2 SD as normal, > +2 SD as overweight.

### Consent

Of the 2,068 PwTB, 1,238 (60%) consented to receive food support (Figure 2). Consent levels differed by sector of notification: 82% (980/1,190) in the public sector versus 29% (258/878) in the private sector. Qualitative findings highlighted several reasons for lower consent in the private sector. Financially secure patients preferred that support be directed to others perceived as more needy, while concerns about confidentiality and social stigma prompted some to avoid enrolment. Programme staff additionally reported that private-sector clinicians often provided limited counselling on the scheme, suggesting an opportunity to enhance consent processes through stronger engagement of private providers. From the programme perspective, the digital consent workflow posed operational challenges: short-duration one-time-passwords, inability to modify consent status once recorded, and mandatory and complicated process of linking consent with ABHA ID. Staff noted that consent status is dynamic and can change over time; they recommended enabling editable consent fields on the Ni-kshay portal. In UPHCs without active donors, staff perceived limited value in recording consent.

**FIGURE 2.**
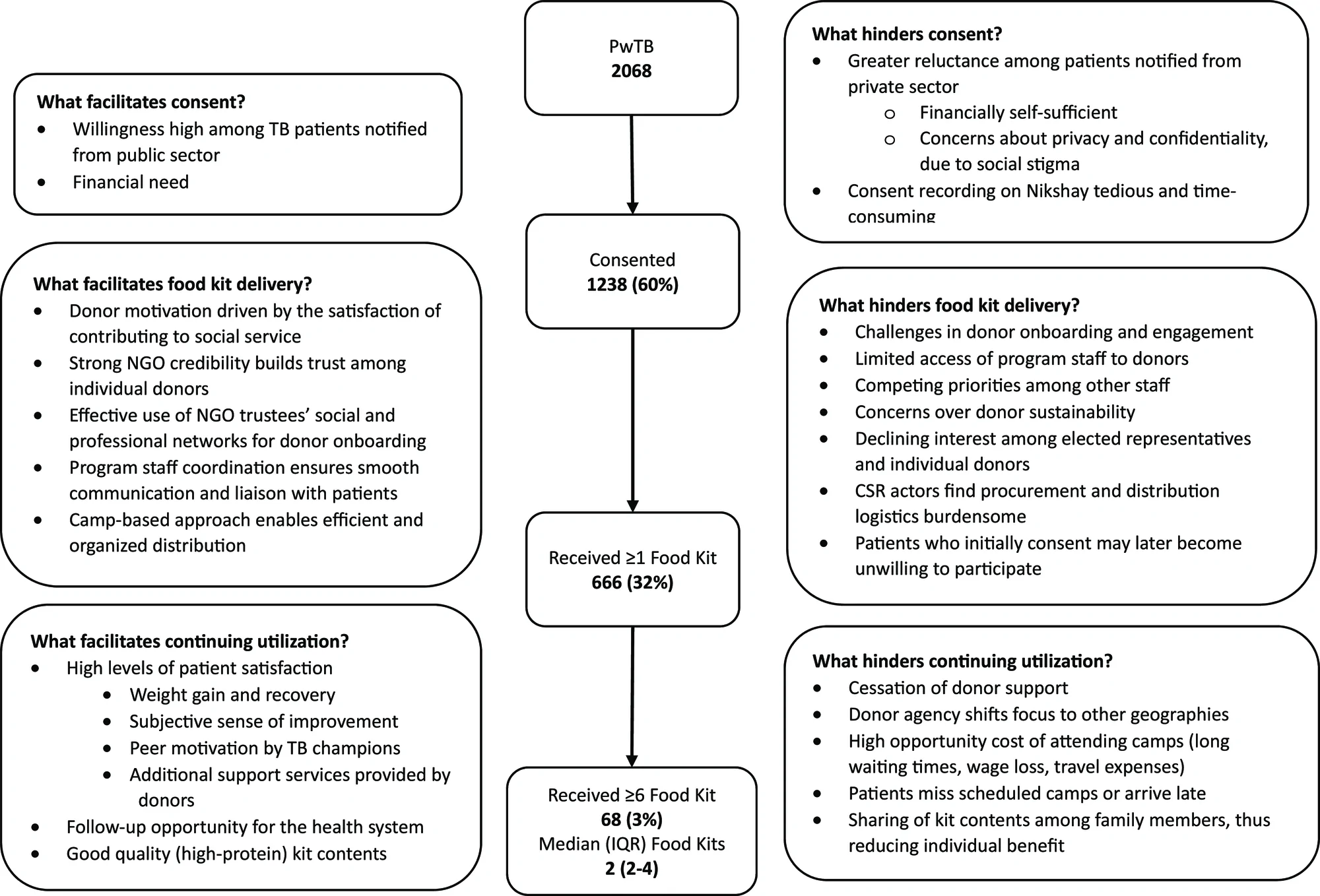
Facilitators and barriers of *Ni-kshay Mitra* coverage among PwTB notified from January to June 2025 in Vadodara City, Gujarat, India.

### Kit receipt

Overall, 666 (32%) PwTB received at least one food kit (Figure 2), but only 68 (3%) received the full course of kits (≥6) throughout the treatment. Higher likelihood of kit receipt was observed among PwTB who were notified through the public sector, residing in north zone, of younger age, residing in slum, consuming tobacco, female, and diagnosed through active case finding (Table 2).

**TABLE 2.** Factors associated with receipt of food kits among persons with drug-sensitive tuberculosis (TB) notified and started on first-line treatment between January and June 2025 in Vadodara City, Gujarat, India.

Characteristics	Categories	PwTB	Received kits^A^	Unadjusted	Adjusted
		N	n (%)^B^	RR (95% CI)	RR (95% CI)
Total		2,068	666 (32)		
Age, years*	0–14	70	23 (33)	1.4 (0.9–2.0)	1.5 (1.1–2.2)
	15–29	660	233 (35)	1.5 (1.2–1.8)	1.3 (1.1–1.6)
	30–44	540	195 (36)	1.5 (1.2–1.9)	1.3 (1.1–1.6)
	45–59	434	128 (30)	1.2 (0.9–1.6)	1.2 (1.0–1.4)
	60 and above	364	87 (24)	1	1
Gender*	Men	1,136	352 (31)	1	1
	Women	930	313 (34)	1.1 (1.0–1.2)	1.2 (1.1–1.4)
	Transgender	2	1 (50)	1.6 (0.4–6.5)	1.7 (0.9–3.0)
BMI categories^C^	Underweight (<18.5 kg/m^2^)	936	375 (40)	1.5 (1.2–1.8)	1.2 (0.9–1.5)
	Normal (18.5–22.9 kg/m^2^)	903	228 (25)	0.9 (0.7–1.2)	0.9 (0.7–1.2)
	Overweight (>23 kg/m^2^)	229	63 (28)	1	1
Zones*^,^ ^D^	East	549	157 (28)	1	1
	North	454	202 (45)	1.6 (1.3–1.8)	1.6 (1.4–1.9)
	South	377	139 (37)	1.3 (1.1–1.6)	1.3 (1.1–1.5)
	West	688	168 (24)	0.9 (0.7–1.0)	0.9 (0.7–1.1)
Urban slum*	No	1,149	267 (23)	1	1
	Yes	919	399 (43)	1.9 (1.6–2.1)	1.4 (1.2–1.6)
Notification*	Public sector	1,190	537 (45)	3.1 (2.6–3.6)	2.5 (2.1–3.0)
	Private sector	878	129 (15)	1	1
Bacteriological confirmation	No	865	246 (28)	1	1
	Yes	1,203	420 (35)	1.2 (1.1–1.4)	0.9 (0.8–1.1)
Case finding	Active	399	184 (46)	1.6 (1.4–1.8)	1.1 (1.0–1.3)
	Passive	1,669	482 (29)	1	1
Site of TB	Pulmonary	1,309	450 (34)	1.2 (1.1–1.4)	1 (0.9–1.3)
	Extra-pulmonary	745	213 (29)	1	1
	Not recorded	14	3 (21)	0.8 (0.3–2.1)	1.2 (0.6–2.7)
Diabetes	No	1834	601 (33)	1	1
	Yes	160	54 (34)	1 (0.8–1.3)	1.2 (1.0–1.5)
	Not recorded	74	11 (15)	0.5 (0.3–0.8)	0.7 (0.3–1.7)
HIV status	Non-reactive	1989	652 (33)	1	1
	Reactive	22	5 (23)	0.7 (0.3–1.5)	0.6 (0.3–1.3)
	Not recorded	57	9 (16)	0.5 (0.3–0.9)	0.9 (0.4–1.7)
Current tobacco use*	No	1,592	470 (30)	1	1
	Yes	387	180 (47)	1.8 (1.4–1.8)	1.3 (1.1–1.6)
	Not recorded	89	16 (18)	0.6 (0.4–1.0)	1.1 (0.6–2.2)
Alcohol use	No	1730	534 (31)	1	1
	Yes	231	106 (46)	1.5 (1.3–1.7)	0.9 (0.8–1.2)
	Not recorded	107	26 (24)	0.8 (0.6–1.1)	1.1 (0.7–1.5)

*RR was statistically significant (*P* value < 0.05).

PwTB = persons with TB; CI = confidence interval; RR = risk ratio; BMI = body mass index.

A Received at least one kit during treatment.

B Row percentage.

C BMI calculated as weight (kg) divided by height (m^2^) for participants aged ≥20 years. For participants aged <20 years, nutritional status was assessed using BMI-for-age *Z*-scores calculated using WHO age- and sex-specific growth standards and clubbed as follows: BMI < −2 SD as underweight, –2 SD to +2 SD as normal, > +2 SD as overweight.

D City is divided into four zones each comprising of two TB units.

Qualitative analysis indicated that kit receipt primarily depended on the donor availability, which varied geographically, because donors were mapped to specific UPHCs. In UPHCs without an active donor, beneficiaries remained uncovered. When supplies were insufficient, medical officers tended to prioritise underweight or economically vulnerable patients.

Programme staff highlighted challenges in onboarding donors due to limited outreach networks among field staff and competing work priorities. Individual donors and elected representatives showed declining interest over time, and CSR contributors found procurement and distribution logistics cumbersome. NGOs were perceived as the most reliable donor category, driven by strong internal motivation, access to donor networks, coordinating team, transparent processes, strong coordination with programme staff, and established community credibility. Monthly camp-based distribution, often conducted at UPHCs in the presence of donors, facilitated smooth and organised kit delivery.

### Continuing utilisation of kits

Median kits received were 2 (interquartile range [IQR]: 2–4) (Figure 2). It was higher in the public sector (median 3; IQR 1–4) compared to the private sector (median 2; IQR 2–4). Beneficiaries described multiple motivations for continued attendance at distribution camps: good kit quality, protein-rich contents, weight gain, perceived improvements in wellbeing, and encouragement from peer supporters such as TB Champions. Donors who provided additional forms of assistance (awareness talks, support for children’s education, blankets in winters) further enhanced participation. Camps also enabled monthly clinical follow-up, offering opportunities for review of symptoms, investigations, and adherence support.

Continuity of support was disrupted for many PwTB after a major donor withdrew mid-way through the cohort’s treatment period. In some cases, donors shifted activities to other geographic areas, leaving certain UPHCs temporarily uncovered. Beneficiary-level barriers included long waiting times at camps, wage loss, travel expenses, missed appointments, or late arrival.

Programme staff observed that the kit contents recommended in the original guidelines made the packages bulky and difficult to carry. After cereals were replaced by high-value items (such as pulses), PwTB found the modified kits more acceptable and convenient.

Sharing of kit contents among family members was a common phenomenon observed. Programme staff attempted to address this through counselling and by increasing the proportion of ready-to-eat items (groundnuts, jaggery, roasted chickpeas, protein/milk powder). Some staff recommended increasing kit quantity to account for family members.

### Treatment outcomes

The proportion with unsuccessful treatment outcomes was 7% in 2022 (pre-scheme) compared to 6% in 2023 (post-scheme) (Table 3). The difference was not statistically significant after adjusting for demographic, clinical, and behavioural characteristics in multivariable analysis (data not shown).

**TABLE 3.** Treatment outcomes among new persons with tuberculosis notified and started on treatment during before (January–June 2022) and after (January–June 2023) implementation of *Ni-kshay Mitra* in Vadodara City, Gujarat, India.

Cohort	Before *Ni-kshay Mitra* (January–June 2022)	*Ni-kshay Mitra* (January–June 2023)
	n (%)^A^	n (%)^A^
Total persons with tuberculosis	1,917 (100)	1,726 (100)
Successful treatment outcome	1,791 (93)	1,631 (94)
Cured	386 (20)	415 (24)
Treatment completed	1,405 (73)	1,216 (70)
Unsuccessful treatment outcome	126 (7)	95 (6)
Treatment failure	7 (0)	5 (0)
Loss to follow-up	14 (1)	10 (1)
Died	95 (5)	68 (4)
Not evaluated	10 (1)	12 (1)

A Column percentage. Some percentages are mentioned as zero because we have rounded off to zero decimals and hence may not add up to 100%.

## DISCUSSION

This mixed-methods study is one of the first comprehensive assessments of the *Ni-kshay Mitra* scheme in an urban Indian setting offering critical insights into its coverage, implementation processes, and potential influence on treatment outcomes among PwTB. By integrating programme data with perspectives from patients, providers, and donors, the study provides practical, action-oriented lessons on how the scheme is functioning in routine settings.

Only about 6 in 10 PwTB consented to receive support. Consent rates were markedly higher in the public sector (83%) compared to the private sector (30%) – this may be due to financial need, differences in patient counselling, trust in public health system, and awareness of social support schemes. Consent rates were higher and ranged from 82% to 97% in other Indian studies.^7,10,13^ This difference is likely influenced by the distinct public–private mix of patients in our setting. Qualitative findings revealed that financially secure individuals in the private sector often declined support in favour of others perceived as more needy. Stigma, fear of confidentiality breaches, and reluctance to be publicly identified as a TB patient were additional deterrents. These concerns have been echoed in earlier qualitative studies on *Ni-kshay Mitra*, where stigma and privacy-related fears reduced patient willingness to enrol.^13^

Overall, one third of PwTB received at least one kit in our study – this was relatively higher compared to an earlier analysis (10%).^7^ Only 3% received the full package of kits in our study which is relatively lower than the study from Puducherry (7%).^13^ Receipt was strongly influenced by factors that were largely structural rather than patient-driven. The most prominent determinant was donor availability, which was uneven across zones because donors were mapped to specific UPHCs. Consequently, PwTB residing in areas without active donors remained uncovered regardless of need or eligibility. This aligns with previous evaluations that reported geographic inequities as a major limitation of the scheme.^7,13^

PwTB notified from the public sector were substantially more likely to receive kits. Other predictors – such as younger age, residence in slum areas, tobacco use, and female sex – reflect patterns of vulnerability or prioritisation observed in frontline decision making. For example, when supplies were limited, medical officers prioritised underweight or economically disadvantaged patients, consistent with the scheme’s goal of addressing undernutrition among the most vulnerable.

Donor characteristics significantly shaped the programme’s functioning. NGOs were the most consistent and reliable contributors, due to established systems and strong internal motivation. In contrast, individual donors and elected representatives often withdrew support over time, while CSR partners struggled with procurement logistics. These findings highlight the need for systematic, long-term donor engagement strategies.

Although kit receipt was inconsistent, PwTB generally valued the support. Many reported improved strength, appetite, and wellbeing. TB Champions and additional support activities (e.g., health talks and seasonal items) increased engagement and satisfaction. However, several barriers hindered regular utilisation. Long waiting times, wage loss, and travel expenses posed major challenges. Donor withdrawal also disrupted continuity. Kit bulkiness initially reduced acceptability, but revisions to more compact contents improved usability.

We did not find any association of Ni-kshay Mitra with treatment outcomes. This may be due to low and inconsistent coverage of the scheme. However, there is existing evidence that nutritional support improves treatment outcomes in PwTB.^3,14^

The study had several strengths. We used a mixed-methods design combining large city-wide programme cohort data with qualitative insights from PwTB, staff, and donors. Multiple data sources enabled triangulation and strengthened credibility. Using routine programme data enhanced operational relevance of findings and reflected real-world conditions. We adhered to ‘Strengthening Reporting of Observational Studies in Epidemiology’ (STROBE) and COREQ guidelines for reporting.^12,15^ The study had some limitations too. Reliance on routine programme data introduced risks of incomplete or delayed entries, particularly for consent and kit delivery. The treatment outcomes were compared using an ecological before–after design, which limits causal inference. As a single city urban study, generalisability to rural or resource limited settings is limited.

The findings suggest several programmatic and policy considerations. Where donor resources are limited, nutritionally vulnerable PwTB – especially those undernourished – should be prioritised to ensure equitable kit distribution. Current geographic mapping of donors to UPHCs restricts need-based allocation, underscoring the importance of flexible systems. Lower uptake among privately notified PwTB raises confidentiality concerns, though many in this group were financially able to contribute as donors post-treatment, offering opportunities for community engagement. Strengthening NGO-driven social media outreach for crowdfunding could expand resources and coverage. Alternative operational models may also improve donor participation. CSR donors, who can support larger cohorts, may be better utilised through NGO channels or e-commerce-based procurement and home-delivery systems to simplify logistics and reduce opportunity costs for patients. Future research should examine these approaches and the feasibility of including ready-to-eat items in the kits.

## CONCLUSION

The *Ni-kshay Mitra* scheme shows strong potential as a community-engagement strategy complementing TB care with nutritional support. Its success depends on equitable coverage, sustained donor contributions, and fewer operational barriers. Strengthening these areas can advance India’s TB elimination goals and inform similar global initiatives.
